# Philadelphia Freedom

**DOI:** 10.5888/pcd9.120182

**Published:** 2012-09-06

**Authors:** James S. Marks, Risa Lavizzo-Mourey

**Affiliations:** Corresponding Author: James S. Marks, MD, MPH, Robert Wood Johnson Foundation, Rte 1 and College Rd East, PO Box 2316, Princeton, NJ 08543. Telephone: 609-627-5796. E-mail: jmarks@rwjf.org.; Author Affiliation: Risa Lavizzo-Mourey, Robert Wood Johnson Foundation, Princeton, New Jersey.

The song “Philadelphia Freedom” became popular in 1976, the bicentennial of our nation’s birth. That was also about the time that the obesity rate in our young people began to rise (1). And it has done so inexorably since then — until now.

Dr. Sam Posner, editor in chief of *Preventing Chronic Disease (PCD)*, had the opportunity to speak with the authors of this week’s publications about their research on the country’s childhood obesity epidemic. Dr. Giridhar Mallya, director of policy and planning for the Philadelphia Department of Public Health, and Dr. James Marks, senior vice president and director of the Health Group at the Robert Wood Johnson Foundation, shared their insights on the obesity epidemic and described the importance of the research featured this week in *PCD*.

Listen to the podcast interview Audio/Video file [MP3 – 5.5MB]

A transcript of this interview is also available. [DOC – 25KB]

That’s what makes the report from Philadelphia so exciting. It’s the latest in a small but growing series of studies that point to the first signs of declining rates of obesity among children in places like New York City and California (2,3). In New York City, declines were seen citywide, but the largest changes were among white and Asian students, who already had the lowest rates (2). In California, the state had a significant overall decline, but progress was uneven. Although counties like Los Angeles, which had been at the forefront of making healthy changes, succeeded in reducing childhood obesity rates, more than half of the state’s counties showed continued increases (3).

Until now, the early reports seemed to portend a tragic victory: we might reverse the slope of the overall epidemic but widen disparities between minority and majority and between rich and poor. The current high rate of obesity and its consequences — disease, disability, and reduced productivity — already are acknowledged as a threat to our nation’s physical and fiscal health (4,5). And as US income inequality continues to grow, health disparities are only likely to worsen. That demands action, and it’s why this report from Philadelphia is so important.

Despite its own economic challenges, Philadelphia was able not only to achieve an overall decline in obesity but also to make the largest improvements among African American male and Hispanic female students (6). There were similar findings among those with severe obesity. There also were statistically significant reductions in obesity rates among students who were eligible for free or reduced-price lunches, although they were not as large as the decline among students who were not eligible for either.

So Philadelphia is a positive deviant, a crucial proof of the concept that communities can reduce obesity rates — and do so in a way that helps to close the disparities gap. We need to learn from the City of Brotherly Love and spread knowledge on the actions and policies that work.

Philadelphia’s report comes on the heels of the Institute of Medicine (IOM) report, *Accelerating Progress in Obesity Prevention* (4). The IOM was clear that the science has progressed to a point where it’s good enough to urge everyone in our society to play his or her part in implementing 5 goals to reverse the obesity epidemic:

- Integrate physical activity into people’s daily lives.
- Make healthy food and beverage options available everywhere.
- Transform marketing and messages about nutrition and activity.
- Make schools a gateway to healthy weights.
- Galvanize employers and health care professionals to support healthy lifestyles.

Philadelphia has sounded the bell, confirming that the IOM is right and calling other cities to action. Get Healthy Philly is a cross-sector, comprehensive effort that

- integrated healthy living and health impact assessments into Philadelphia 2035, the city’s new comprehensive plan;
- increased access to healthy foods for 220,000 residents of low-income neighborhoods;
- installed nearly 30 miles of bicycle lanes;
- increased parents’ awareness about the sugar content of beverages like soda, fruit drinks, and sweet teas through a media-education initiative that was seen or heard more than 40 million times;
- established active School Wellness Councils in 171 public schools serving more than 100,000 students to incorporate physical activity into the school day and remove junk foods from classrooms, school stores, and fundraisers;
- implemented nutrition and physical activity standards for more than 300 afterschool and recreation programs that serve 20,000 children annually; and
- partnered with local businesses to increase worksite wellness.

The birthplace of our nation’s liberty is becoming a cradle of good health by implementing policies strong enough to overcome the barriers to health that many of our cities face (7).

In 2007, when the Robert Wood Johnson Foundation committed $500 million toward the goal of reversing the childhood obesity epidemic by 2015, our commitment followed closely on an earlier IOM report, *Preventing Childhood Obesity: Health in the Balance*. That report signaled that the actions underway to reverse the epidemic then were too few, too small, too fragmented, and too weak to succeed (8). And the science was immature. We’re in a different place today.

The IOM now concludes that the science has matured and provides strong evidence for how to reverse the epidemic. The results from Philadelphia make it clear that, if we act using what we know, we will succeed. So now the fundamental question is: do we have the will to act?

The Philadelphia study could not come at a better time as our nation struggles with skyrocketing medical care costs. Recent estimates forecast much higher rates of obesity and related costs, but also suggest that, if we could keep obesity rates at 2010 levels, we could save $550 billion dollars in medical care costs over the next 20 years (9). Philadelphia is pointing the way to a path for doing just that.

The biggest surprise is how far we’ve come as a nation in combatting the epidemic in such a short period of time. In July 1776, our Founding Fathers gathered in the city to pen the Declaration of Independence, and in December of that year, we turned the corner in our war for independence just across the river from Philadelphia. Turning the corner was not the end of that war effort. There were many more tough days ahead, and such will be the case with the obesity epidemic. Nevertheless, victories give hope. And we definitely have hope.

Good health is crucial to the pursuit of happiness called for in the Declaration of Independence. For our nation’s young people to have equal opportunity to pursue happiness, they also must have equal opportunity for good health, regardless of where they live. Philadelphia’s success, early and fragile though it is, indicates that we can overcome the obesity epidemic in a way that strengthens our nation as a whole and helps us realize our most profound aspirations as a people.
