# Supplementary material for: Philadelphia Freedom
Source: Prev Chronic Dis. 2012 Sep 6;9:E144. doi: 10.5888/pcd9.120182 (PMC3475517; doi:10.5888/pcd9.120182)
Supplement: Supplementary file 2 [file 12_0118_01.doc]

# Childhood Obesity in Philadelphia

[Sam Posner] Thank you for joining us today. On the phone with me is Dr. Giridhar Mallya, director of policy and planning for the Philadelphia Department of Public Health and Dr. James Marks, the senior vice president and director of the Health Group at the Robert Wood Johnson Foundation. Dr. Mallya is the author of an article recently published in CDC’s e-journal Preventing Chronic Disease, entitled “Obesity and Severe Obesity among Students in the School District of Philadelphia: Prevalence, Disparities, and Trends, 2006 to 2010.” Dr. Mallya, please briefly describe the impetus for conducting this study and give a brief summary of the key findings.

[Giridhar Mallya] What we found is that using annual height and weight data on approximately 120,000 Philadelphia school children. We found that the prevalence of obesity decreased from 21.5% to 20.5% between 2006 and 2010. To put it in perspective, that’s actually a statistically significant 5% decrease over that time period. There were also significant decreases among elementary and middle school-aged kids, but not high schoolers. And probably the most encouraging thing was that we saw a significant 8% decrease in obesity among African American boys and a 7% decrease among Hispanic girls.

[Sam Posner] Great, thank you. Dr. Marks, the Robert Wood Johnson Foundation has been at the forefront of the movement to try and end the childhood obesity epidemic. The Foundation has committed $500 million toward the goal of reversing the childhood obesity by 2015. Do you think that the decline is really meaningful and what do you think it will take to take this to scale and really decrease childhood obesity across the nation?

[James Marks] Well, it is meaningful very clearly—both statistically significant and also as a proof of concept. It’s not large enough to get us back to where we were 30 years ago, but we really hope it signals a turning point. I’ve mentioned we’ve seen other communities with declining rates, but not enough. We need to see these changes nationwide. So, the federal surveys that look at the whole nation don’t yet show a decline. But we’re hopeful that as more and more communities make changes like Philadelphia and some of the others have made that we will see these through the nation as a whole. No single intervention is going to turn this around. It really takes a lot of changes like Philadelphia has made, so it’s not just going to be sugary drinks out of schools, though that’s really important. It’s not just going to be shifting to lower fat milk, though that’s important, but each of these small steps is what will add up to the kind of changes we need. And I think that’s very important because you’re almost always asked, well if you had to do one thing, what would it be? And the answer is, you’ve got to do it all.
